# Subliminal conditioning of vestibular perception generalizes within otolith organs

**DOI:** 10.1007/s00415-022-10982-7

**Published:** 2022-02-03

**Authors:** Aram Keywan, Gharam Yassin, Klaus Jahn, Max Wuehr

**Affiliations:** 1 German Center for Vertigo and Balance Disorders, University Hospital of Munich, Munich, Germany; 2 Schön Klinik Bad Aibling, Bad Aibling, Germany

Dear Sirs,

Previous studies in animals and humans provide evidence that the vestibular system is capable to autoregulate its sensitivity in response to prolonged exposure to either high- or low-amplitude vestibular stimuli. In an aquatic animal model, the gain of the vestibulo-ocular reflex (VOR) was shown to decrease or increase when being exposed to sustained passive oscillatory head motion at high or low amplitudes, respectively [6]. Analogous effects were demonstrated in humans, where prolonged exposure to high-intensity passive motion attenuates vestibular-mediated balance responses and increases thresholds for vestibular motion perception [7]. We recently complemented the latter finding by showing that human perceptual thresholds can conversely be decreased in response to long-lasting imperceptible passive head oscillations—a phenomenon we designated as subliminal vestibular conditioning [10]. Taken together, such bi-directional adaptive re-calibrations within vestibular information processing resemble the characteristics of a homeostatic plasticity [6] that is able to counterbalance prolonged elevated or diminished neuronal activity to stabilize vestibular encoding around a certain set point of activity [14].

Subliminal conditioning of vestibular perception is likely mediated by plasticity within central vestibular networks. Surgical ablation of the cerebellum in animal models has accordingly been shown to prevent homeostatic plasticity within the VOR circuitry [6]. Other central mechanisms such as adaptive plasticity at the synapse between vestibular afferents and first-order central neurons may further mediate homeostatic adaptions in vestibular signal processing [11]. Since inputs from the different vestibular end organs, i.e., otolith and semicircular canal (SCC) receptors, show considerable central convergence [4, 5, 13, 15], it is thus conceivable that the sensitizing effects of subliminal vestibular conditioning are not specific for the particular motion plane being stimulated but rather generalize over other non-stimulated structures of the vestibular periphery.

We previously tested this hypothesis by examining whether conditioning-induced sensitization of otolith-mediated perception may also affect SSC-mediated perceptual thresholds but did not find evidence for such crosstalk [10]. The purpose of this follow-up study was to examine whether alternatively a conditioning-induced crosstalk may exist between the two otolith organs. To this purpose, we tested if translational passive motion conditioning along the horizontal interaural axes, which sensitizes utricular-mediated perception, also affects saccular-mediated perception for translations along the earth-vertical axis.

**Fig. 1:**
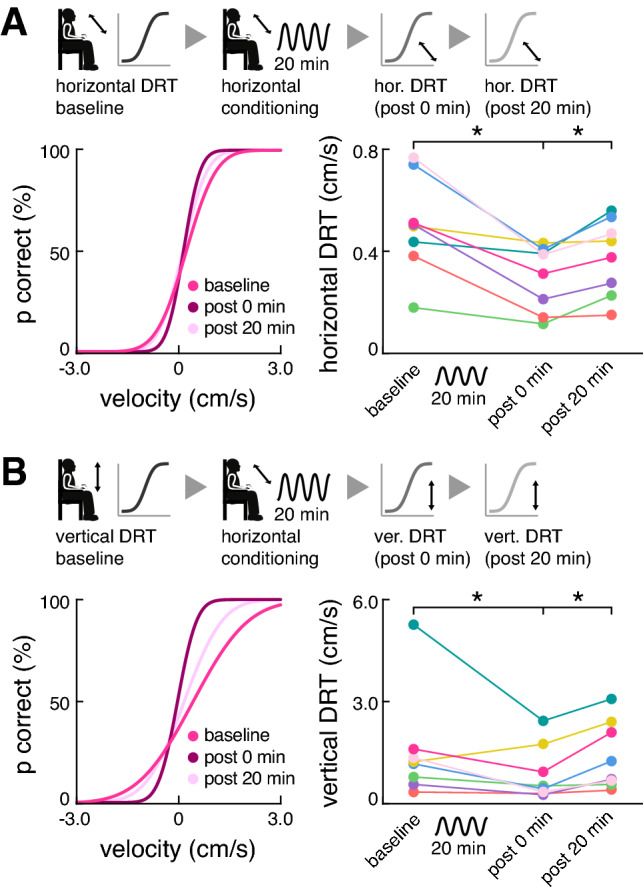
Conditioning effects on horizontal and vertical vestibular perceptual thresholds. Within- and cross-plane conditioning effects were tested in two separate sessions. **A** Upper panel: experimental procedure for testing within-plane effects of horizontal translational conditioning on utricular-mediated horizontal translational DRTs; lower panel: exemplary psychometric curve fits (left) and group results (right) of the perceptual performance for horizontal translational motion stimuli immediately before (baseline), immediately after (post 0 min), and 20 min after (post 20 min) horizontal conditioning. **B** Upper panel: experimental procedure for testing cross-plane effects of horizontal translational conditioning on saccular-mediated vertical translational DRTs; lower panel: exemplary psychometric curve fits (left) and group results (right) of the perceptual performance for vertical translational motion stimuli immediately before (baseline), immediately after (post 0 min), and 20 min after (post 20 min) horizontal conditioning. *DRT* direction recognition threshold

Eight healthy subjects (four males, mean age 27.1 ± 2.1 years) that were naive to the experimental protocol participated in the study. None of the participants reported any auditory, vestibular, or cardio-vascular disorders. All were familiar with the procedures of vestibular threshold testing from previous studies and gave their written informed consent prior to the experiments. The experimental procedures were analogous to our previous study [10]. Shortly, the experiment consisted of two parts separated by a resting period of one hour. In the first part, the effects of translational passive motion conditioning along the earth-horizontal interaural axis on utricular-mediated perception was tested (Fig. 1A). In the second part, potential crosstalk between the horizontal conditioning stimulus and saccular-mediated perception for translations along the earth-vertical superior-inferior axis was tested (Fig. 1B). For both experiments, vestibular direction-recognition thresholds (DRT) at 1 Hz were determined immediately before (baseline), immediately after (post 0 min), and 20 min after (post 20 min) conditioning while participants were seated on a 6DOF motion platform (Moog, 6DOF2000E, East Aurora, New York; 150 trials each following a 3-down 1-up paradigm [3, 8]). DRTs were determined in darkness with eyes closed and participants wore noise-canceling headphones to mask any sound cues resulting from the platform motion.

The subliminal conditioning stimulus consisted of a 1 Hz sinusoidal translational motion along the horizontal interaural axis. Conditioning was applied for a total duration of 20 min while participants were quietly sitting on the motion platform with their head stabilized in an upright, straight-ahead position and eyes closed [6, 10]. For each participant, the conditioning stimulus amplitude was individually adjusted to 70% of their baseline vestibular perceptual threshold for horizontal translations and consequently remained below perceptual level. Statistical comparisons were performed on the log-transformed DRTs to achieve normal distribution [8]. The effects of horizontal conditioning on utricular- and saccular-mediated DRTs were evaluated by a repeated-measures ANOVA and Bonferroni post hoc analysis with the factor session (baseline, post 0 min, post 20 min) using SPSS (version 26.0, IBM corp., Armonk, NY).

Baseline assessment of vestibular DRTs yielded an average of 0.50 ± 0.19 cm/s for horizontal interaural and 1.51 ± 1.57 cm/s for vertical translational motion. The individual conditioning stimulus was accordingly set to 70% of the baseline horizontal DRT (in average 0.35 ± 0.13 cm/s peak velocity) and not perceived by any of the participants. Horizontal translational conditioning significantly lowered horizontal DRTs in all participants by in average 39 ± 19% (post 0 min vs. baseline: F_1,7_ = 44.3, *p* = 0.006; Fig. 1A). Horizontal DRTs had returned to baseline level 20 min after conditioning (post 20 min vs. post 0 min: F_1,7_ = 44.3, *p* = 0.016). Remarkably, the effects of horizontal conditioning on vertical DRTs were found to be more or less analogous. Immediately after conditioning, vertical DRTs were significantly lowered for all except one participant by in average 35 ± 35% (post 0 min: vs. baseline: F_1,7_ = 17.2, *p* = 0.041; Fig. 1B) and had returned to baseline 20 min after conditioning (post 20 min vs. post 0 min: F_1,7_ = 17.2, *p* = 0.016).

These results first confirm our previous observation that an exposure to prolonged low-intensity oscillatory head motion can induce sensitization of the vestibular perceptual capacity in healthy adults [10]. In agreement with prior observations in animal models and humans, we found that the conditioning-induced lowering of perceptual thresholds was transient with a relatively short time constant and had reversed already 20 min after cessation of the conditioning stimulus. This observation further complements previous reports on analogous transient adaptions of sensitivity in human visual, auditory, and somatosensory perception induced by prolonged subliminal sensory stimulation [1, 2, 16].

Notably, we found that passive translational motion conditioning along the horizontal interaural axis equally sensitized utricular-mediated perception with a sensory epithelium aligned to the conditioning movement and saccular-mediated perception with a sensory epithelium orthogonal to motion direction. Although we cannot exclude a small amount of co-activation of saccular afferents during utricular conditioning (due to variations in head orientation and/or undirected vibrational noise induced by the motion platform [3]), this would most likely not suffice to induce sensitizing effects of the same order of magnitude (threshold reduction of 39% for utricular- vs. 35% for saccular-mediated perception).

Alternatively, we propose that the generalization of conditioning effects over non-stimulated directions of motion is rather of central origin. Utricular and saccular otolith afferents are commonly tuned to respond to a particular preferred direction vector. In contrast, central vestibular nuclei that frequently receive convergent inputs from both otolith organs typically exhibit a multi-dimensional tuning and equally respond to motion along two or even three orthogonal direction vectors [4, 5]. Hence, homeostatic plastic changes of neuronal encoding within central vestibular nuclei neurons could mediate adaptions of vestibular sensitivity for both stimulated was well as non-stimulated orthogonal axes of motion.

In contrast to our present findings, previous reports, however, indicated that conditioning-induced adaptions in vestibular sensitivity are rather specific for the plane being stimulated [6, 10]. Accordingly, our preceding study did not provide evidence that sensitizing effects of utricular-conditioning also affect horizontal-SSC-mediated vestibular perception [10]. One reason for this apparent discrepancy could be the less pronounced central convergence pattern of utricular and horizontal SSC afferents compared to that of afferents from the two otolith organs [15]. Furthermore, prolonged horizontal passive head rotations were found to only induce homeostatic adaptions of the horizontal but not the vertical rotational VOR [6]. This observation was analogously attributed to a rather infrequent and weak convergence of afferent inputs from different SCC along the central VOR circuitry [13].

In conclusion, this study demonstrates that vestibular perception can be transiently sensitized by a prolonged subliminal stimulation with imperceptible oscillatory head motion. This effect not only targets the stimulated sensory end organ structures but may at least partly generalize over other non-stimulated receptors of the vestibular periphery. In such a way, subliminal vestibular conditioning may be used therapeutically to boost previously suggested autoregulatory processes in the vestibular system [9, 12] to treat decrements of vestibular sensitivity in the elderly and patients with uncompensated vestibular hypofunction. A practical implementation of subliminal vestibular conditioning in a rehabilitation context may be accomplished by for instance prolonged standing on a balance board or sitting on an exercise ball. However, since the here reported conditioning-induced changes in vestibular sensitivity were transient and receded with a relatively short time constant, further research is required to examine whether longer lasting or repeated conditioning may yield temporally extended homeostatic adaptions of vestibular sensitivity.
